# New paths for *PLOS Neglected Tropical Diseases*: Continuing a tradition of innovation and commitment to the poor

**DOI:** 10.1371/journal.pntd.0005862

**Published:** 2017-10-26

**Authors:** Peter J. Hotez

**Affiliations:** Texas Children’s Hospital Center for Vaccine Development, National School of Tropical Medicine, Baylor College of Medicine, Houston, Texas, United States of America

In 2017, *PLOS Neglected Tropical Diseases* marked its 10th anniversary. As highlighted during a celebratory anniversary event held at the World Health Organization in Geneva, *PLOS Neglected Tropical Diseases* was founded to coincide with the shaping and creation of the modern framework of neglected tropical diseases (NTDs) [1–3]. It was intended to give the first open access voice to a community of scientists and public health experts working hard to control and eliminate NTDs in the world’s poorest countries and areas, as well as to those designing and conducting experiments to gain fundamental insights into the pathogenesis of NTDs and the development of new drugs, vaccines, diagnostics, and biocides. We also tried to champion the voice of those asking tough and daunting social science and policy questions about the NTDs.

Some of our “big-picture” metrics and achievements were highlighted previously [3]. They include our editorial board, which is highly represented (40%) by editors from disease-endemic countries, and almost 5,000 published papers with at least one-quarter of the authors each hailing from Africa, Asia, and Latin America, respectively [3]. Our papers are highly cited with article-specific metrics, indicating that many of our papers have been viewed by tens of thousands of individuals and 1 paper more than 200,000 times [3]. We’re also able to follow how *PLOS Neglected Tropical Diseases* articles are tracked on social media and highlighted in the world’s most widely circulating newspapers. Many of our papers have influenced the global policy makers from both disease-endemic nations and donor countries as well as WHO and other major international health agencies. Overall, our journal has raised awareness about the NTDs and played an important role in promoting access to NTD medicines and treatments as well as access to innovation by supporting research and development on these diseases.

It has been a great honor for me to help lead efforts that launched the founding of *PLOS Neglected Tropical Diseases*, and together with my friend and colleague Professor Serap Aksoy, to guide the journal through its first decade. There is no question that *PLOS Neglected Tropical Diseases* has been one of my greatest passions, and it’s been a fulfilling experience serving as Co-Editor-in-Chief (EIC).

Like with any important journal, I also think it’s wise to refresh and look to new leaders who can shepherd *PLOS Neglected Tropical Diseases* through its next journey. Beginning in January 2018, I will transition away from day-to-day journal management in order to serve as the founding EIC. In that role, I hope to continue contributing editorials and viewpoints for *PLOS Neglected Tropical Diseases*, providing overall guidance for future directions, and representing our journal at future summits and meetings when asked to do so. I also hope to continue submitting research articles on our NTD vaccine development program.

Professor Judd Walson will now join Professor Aksoy as Co-EIC. Many in the NTD community already know Professor Walson through his clinical trial and implementation research in infectious disease or in his current role as Deputy EIC. I want to thank Professor Walson for agreeing to take on the role of Co-EIC as I transition to founding EIC. There continue to be many opportunities to innovate and improve biomedical publishing, particularly as it relates to diseases of the poor. I expect and hope that *PLOS Neglected Tropical Diseases* will continue to remain at the vanguard of new approaches for peer review and open access. I’m excited to see how our journal progresses as it takes on new and innovative approaches and projects, and I welcome the opportunity to help it move into its second decade!

## References

[1] Hotez P. A new voice for the poor. PLoS Negl Trop Dis. 2007 10 31;1(1):e77 doi: 10.1371/journal.pntd.0000077 17989790 10.1371/journal.pntd.0000077 PMC2041819

[2] Hotez P, Aksoy S. PLOS Neglected Tropical Diseases: Ten years of progress in neglected tropical disease control and elimination … More or less. PLoS Negl Trop Dis. 2017 4 20;11(4):e0005355 doi: 10.1371/journal.pntd.0005355 28426662 10.1371/journal.pntd.0005355 PMC5398476

[3] Hotez P, Bundy DAP. The PLOS Neglected Tropical Diseases decade. PLoS Negl Trop Dis. 2017 4 20;11(4):e0005479 doi: 10.1371/journal.pntd.0005479 28426658 10.1371/journal.pntd.0005479 PMC5398526

